# *PGAP3* Associated with Hyperphosphatasia with Mental Retardation Plays a Novel Role in Brain Morphogenesis and Neuronal Wiring at Early Development

**DOI:** 10.3390/cells9081782

**Published:** 2020-07-27

**Authors:** Sahar I. Da’as, Waleed Aamer, Waseem Hasan, Aljazi Al-Maraghi, Alya Al-Kurbi, Houda Kilani, Jehan AlRayahi, Khaled Zamel, Mitchell A. Stotland, Khalid A. Fakhro

**Affiliations:** 1 Department of Human Genetics, Sidra Medicine, Doha 26999, Qatar; sdaas@sidra.org (S.I.D.); waamer@sidra.org (W.A.); whasan@sidra.org (W.H.); aalmaraghi@sidra.org (A.A.-M.); aalkurbi2@sidra.org (A.A.-K.); 2 Division of Plastic and Craniofacial Surgery, Sidra Medicine, Doha 26999, Qatar; hkilani@sidra.org (H.K.); mstotland@sidra.org (M.A.S.); 3 Division of Pediatric Neurology, Sidra Medicine, Doha 26999, Qatar; jalrayahi@sidra.org (J.A.); kzamel@sidra.org (K.Z.); 4 Department of Genetic Medicine, Weill Cornell Medical College, Doha 24144, Qatar; 5 College of Health and Life Sciences, Hamad Bin Khalifa University, Doha 34110, Qatar

**Keywords:** hyperphosphatasia mental retardation syndrome 4 (HPMRS4), post-GPI attachment to proteins 3 (*PGAP3*), neurological disorder, human disease model, zebrafish, neural tube defect, whole genome sequencing

## Abstract

Recessive mutations in Post-GPI attachment to proteins 3 (*PGAP3*) cause the rare neurological disorder hyperphosphatasia with mental retardation syndrome 4 type (HPMRS4). Here, we report a novel homozygous nonsense mutation in *PGAP3* (c.265C>T-p.Gln89*), in a 3-year-old boy with unique novel clinical features. These include decreased intrauterine fetal movements, dysgenesis of the corpus callosum, olfactory bulb agenesis, dysmorphic features, cleft palate, left ear constriction, global developmental delay, and hypotonia. The zebrafish functional modeling of PGAP3 loss resulted in HPMRS4-like features, including structural brain abnormalities, dysmorphic cranial and facial features, hypotonia, and seizure-like behavior. Remarkably, morphants displayed defective neural tube formation during the early stages of nervous system development, affecting brain morphogenesis. The significant aberrant midbrain and hindbrain formation demonstrated by separation of the left and right tectal ventricles, defects in the cerebellar corpus, and caudal hindbrain formation disrupted oligodendrocytes expression leading to shorter motor neurons axons. Assessment of zebrafish neuromuscular responses revealed epileptic-like movements at early development, followed by seizure-like behavior, loss of touch response, and hypotonia, mimicking the clinical phenotype human patients. Altogether, we report a novel pathogenic *PGAP3* variant associated with unique phenotypic hallmarks, which may be related to the gene’s novel role in brain morphogenesis and neuronal wiring.

## 1. Introduction

Post-GPI attachment to the proteins 3 (*PGAP3*) gene, encoding a Glycosylphosphatidylinositol (GPI)-specific phospholipase, plays a critical role in the biosynthesis of GPI-anchored proteins (GPI-APs). It is ubiquitously expressed and is essential for lipid remodeling through the GPI-APs maturation by the removal of unsaturated fatty acids, a vital step for protein sorting and trafficking [1]. Mutations in *PGAP3* result in hyperphosphatasia with the mental retardation-4 disorder (HPMRS4, OMIM # 615716), a rare autosomal recessive neurologic disorder characterized by structural brain anomalies, severely delayed psychomotor development, mental retardation, hypotonia, seizures, lack of speech acquisition, and dysmorphic facial features. To date, less than 50 patients have been reported worldwide [2,3,4], typically presenting with brain anomalies, including a thin corpus callosum, abnormalities in the periventricular zones, ventriculomegaly, cerebellar hypoplasia, and white matter loss [2,5,6]. One of the hallmarks of HPMRS4 is agenesis or hypoplasia of the corpus callosum affecting the connection between the right and left cerebral hemispheres of the brain [3,7]. Despite this knowledge, the exact cellular and molecular abnormalities at the earliest stages of brain morphogenesis and the disease-associated phenotypes remain mostly obscure.

In mouse models, a complete loss of GPI anchors leads to embryonic lethality [8], precluding its use as a model to explore the origins of disease; however, this could be overcome by generating transient knockdown by morpholino technology to assess near-ablation of protein levels, offering the zebrafish model for recapitulating the phenotypes of different human neurodevelopmental disorders [9,10]. Further, the zebrafish model is a powerful genetic system for the investigation of pathogenicity of candidate human disease genes, offering real-time phenotypic read-outs in transparent developing embryos, and—in the case of neurological conditions—in vivo visualization of the developing nervous system. 

In particular, the zebrafish model offers a high degree of neurological and behavioral resemblance to mammals due to highly conserved brain structures, functions, and neurochemistry, e.g., zebrafish share parallel brain anatomy including a hippocampus, diencephalon, tectum, and cerebellum that has similar cell types and differentiation pathways as in mammals [11,12,13]. Furthermore, the zebrafish brain has a similar organization to the human brain’s three main parts: The cerebrum, cerebellum, and brainstem [14]. The functional homology of the zebrafish cerebellum during visual-motor behaviors is highly conserved [9]. Specifically, in zebrafish, the embryonic forebrain is responsible for sensory processing and input and directing behavior, whereas the smaller midbrain is essential for vision and hearing. It contains the visual processing and response center, the tectum, that is connected by retinal ganglion cells to the retina. The tectum and its connecting neurons are critical for survival, as it is the startle and reflex response center. Finally, the hindbrain incorporates the cerebellum, responsible for motor control, receiving and processing sensory stimuli, and learning. 

Additionally, the midbrain–hindbrain boundary has motor neurons that control the movement of the eyes, jaw, head, and body [11]. It is well established that zebrafish midbrain–hindbrain boundary is essential for sensory responses processed from the developing tectum of the midbrain that has connecting neurons involved in integrating visual and motor inputs and the developing cerebellum of the hindbrain that has the motor neurons [11]. 

In this study, we present the unique case of a child with global developmental delay and brain anomalies and neuromuscular abnormalities. By using whole-genome sequencing, we identify a novel pathogenic *PGAP3* variant that supports a diagnosis of HPMRS4 syndrome, with an expanded clinical presentation. Given the paucity of functional models for this gene, we use zebrafish to investigate the in vivo function of *PGAP3* in brain morphogenesis at the earliest stages of the central nervous system development. Knockdown of *PGAP3* suggested a novel role of this gene during neural tube formation, cerebral ventricular morphogenetic expansion, and neuronal wiring. Our functional studies of the human-associated gene in the zebrafish model provides a mechanistic basis for how *PGAP3* regulate brain formation and suggests a novel, essential role for *PGAP3* in the complex coordination between growth and neuronal wiring during brain development. 

## 2. Methods

### 2.1. Study Subjects

The institutional review board approved this study of Sidra Medicine, Qatar (IRB#-1610004943). The proband and family members were enrolled in this study after obtaining written informed consent. Clinical assessment of the patient was performed by attending physicians from plastic and craniofacial, neurology, and medical genetics. 

### 2.2. Whole-Genome Sequencing

Genomic DNA (gDNA) was isolated from the peripheral blood of the subjects using DNeasy^®^ Blood, and a tissue kit was used (Qiagen sciences LLC, Germantown, MD, USA). 

Whole genomes were on the Illumina Hiseq-X platform using 150 bp paired-end design. The raw sequencing reads were mapped to the reference genome (GRCh37, hg19) using BWA (version 0.7.15 [15]), and genetic variants were called with HaplotypeCaller of the GATK suite (v.4.0) [16]. TheVariant Call Format (VCF) file, containing variants, was annotated using SnpEff v.4.3 [17] then filtered for the candidate, fitting the following criteria: (1) Being in the coding region including exonic, splice-site region, (2) being rare (<1%) in all mutation databases (i.e., 1000 genomes [18], gnomAD [19], ExAC [20], and ESP6500 [21], and (3) co-segregate with the phenotype in the family and follow a specific mode of inheritance (for example, autosomal recessive). The final list of variants was prioritized based on the literature search and whether any of the associated genes have been linked to the patient symptoms, such as known Mendelian genes (https://omim.org).

### 2.3. PGAP3 Protein Homology 

Protein alignment for the human PGAP3 protein (NP_001278659) was performed with PGAP3 orthologs from multiple species, including rat (NP_001137367), mouse (NP_001028709), xenopus (NP_001072247), zebrafish (NP_001108063), and drosophila (NP_610223.1). 

### 2.4. Zebrafish Care and Husbandry 

Zebrafish (*Danio rerio*) wild-type line (AB strain) adults were maintained in a recirculating aquaculture system under standard environmental conditions of temperature at 27 °C, conductivity at 800 µs and pH at 7.5 with 14 h light and 10 h dark cycle. All protocols used in these studies were approved by the local Animal Care and Use Committee and conform to the Zebrafish Policy published by the Qatar Ministry of Public Health that follows the Guide for the Care and Use of Laboratory Animals published by the National Institutes of Health. (http://www.qu.edu.qa/static_file/qu/research/documents/qu_iacuc/MOPH%20Policy%20on%20Zebrafish.pdf). Experiments performed on zebrafish were approved by the IACUC Office of Qatar University (QU-IACUC 26-2/2018-REN1). For zebrafish injection experiments, embryos were collected in *N*-phenylthiourea (PTU) (Sigma, P7629) media and used for microinjection and raised in 28 °C incubators. Larvae at 72 h post-fertilization (hpf) were used for phenotypic examination and imaging for morphological analysis. Zebrafish larvae were euthanized by the administration of Tricaine MS-222 (Western Chemical, 1029D11) anesthetic agent (200 mg/L) followed by chilling on ice. Upon euthanasia, zebrafish carcasses were disposed of as pathological medical waste.

### 2.5. RNA Extraction and Reverse Transcription

Embryos were allowed to develop at 28.5 °C, collected and stored at −80 °C freezer at timed intervals post-fertilization, as specified in figure legend (Figure 2D). Frozen embryos/larvae were homogenized in Trizol Reagent (Ambion, Life technologies, 15596026, Austin, TX, USA) and total RNA extracted from zebrafish at different selected developmental stages, according to the manufacturer’s instructions (Purelink RNA mini kit, cat. 12183018A, Ambion, Life technologies, Austin, TX, USA). Extracts were treated with Turbo DNase (Ambion, Life technologies, 2238G2, Austin, TX, USA) to minimize genomic DNA contamination. The concentration and quality of RNA were determined spectrophotometrically. Reverse transcription of 3 μg of total RNA (20 μL reaction volume) was performed using gene-specific primers with Superscript III first-strand synthesis (Invitrogen, 18080051): Fw: CTGGACGTGTCGTGATGACT, Rev: AAACATAGGAAACGGGCAAA, product size 231 targeting exon 2–3 of the zebrafish *pgap3* transcript- ENSDART00000152737.3. Conventional quantitative real time PCR with the reverse transcribed cDNA template from total RNA was used and ef1a gene (ENSDARG00000020850) was utilized as a housekeeping gene Fw: GAGGAAATCACCAAGGAAGTCAG, Rev: TTGAACCAGCCCATGTTTGAG).

### 2.6. Whole-Mount In Situ Hybridization of the Zebrafish Embryos

Whole-mount in situ hybridization (WISH) in zebrafish embryos was performed as previously reported [22]. The DNA template for zebrafish *pgap3* was amplified from the cDNA of WT embryos at 72 hpf with primers: Fw: CTGGACGTGTCGTGATGACT and Rev with T7: CAGTGAATTGTAATACGACTCACTATAGGGAGAGTAGATGGAGTAGAGAATG, amplicon length 361 bases. Fluorescein isothiocyanate (FITC)-labeled antisense probe was in vitro transcribed by a T7 RNA polymerase kit (Roche) and purified with NucAway spin columns (Invitrogen). Embryos for WISH were prepared by fixing with 4% paraformaldehyde in 1× PBS solution and treated with proteinase K. The antisense probe was hybridized with the fixed embryos at each developmental stage in hybridizing solution. 

### 2.7. Design of Morpholino Antisense Oligos and Zebrafish Microinjection

Translational blocking Morpholino (MO) sequence designed to target the Zebrafish NM_001114591.1 was synthesized by Gene Tools (Corvallis, OR, USA). Zebrafish *pgap3*-MO was designed targeting the ATG start codon of transcripts ENSDART00000091519.4 and ENSDART00000152737.3. MO antisense oligos were dissolved to a final concentration of 2.0 µM. Injections were performed at the 1-cell stage using a PLI-100 Picolitre injector, Harvard Apparatus, as described previously. Optimal doses for the MO were 3, 4.5, and 6 ng. At 24 hpf, the embryos were visualized using a stereomicroscope (Zeiss). To confirm the specificity of the effects of the *pgap3* MO, a second P53 morpholino (p53 MO) was co-injected. For this experiment, the dosage of injected P53 MO was 3.0 ng. Sequences of the *pgap3* MO were MO: 5′GGCCGCGAGAAACATCAGGCCATTA3′-FITC, Standard Control MO: 5′ CCTCTTACCTCAGTTACAATTTATA3′-FITC, p53 MO: 5′ GCGCCATTGCTTTGCAAGAATTG3′. 

### 2.8. Microscopic Observation of Zebrafish Embryos

Zebrafish morphology was examined at 24 hpf, 48 hpf, and 72 hpf, and images of head phenotypes were captured by Stereomicroscope Zeiss LUMAR.V12. The tested zebrafish larvae were sorted into an individual well in a 28 °C incubator. Zebrafish larvae were mounted in 3% methylcellulose for stabilization throughout imaging time. The different zebrafish groups were examined for developmental or teratogenic defects, and if they had any, these larvae were excluded from the analysis. To rule out off-target and nonspecific effects of the *pgap3* MO injection on the expected brain phenotypes, the p53 MO co-injected group was examined as well (data not shown).

### 2.9. Western Blot 

Total protein was extracted from embryos of the control or *pgap3* MO-injected groups (~30 embryos per group) at 72 hpf using an RIPA lysis buffer (Thermo-scientific, 89900) containing phosphatase inhibitor cocktail and halt protease. Zebrafish larvae were homogenized and kept on ice for 30 min. Standard protein electrophoresis protocol was performed as described previously [23]. After electrophoresis, the gel was transferred into a nitrocellulose membrane and incubated with polyclonal anti-PGAP3 antibody (Origene, TA342058), (1:300 dilution) overnight at 4 °C, followed by incubation with rabbit anti-human α-tubulin antibody (Abcam, ab4074), (1:1000 dilution) for 1 h at room temperature. For protein band visualization, the ECL chemiluminescence detection kit (Thermofisher Scientific, 34096, Waltham, MA, USA) was used. Band intensity analysis was performed using ChemiDoc MP Imager (BioRad) supplied with Image Lab software to normalize *pgap3* morphants protein bands to control band. 

### 2.10. Phalloidin Staining of Zebrafish Larvae 

To facilitate imaging, MO-FITC injected zebrafish groups at 72 hpf were rinsed in phosphate-buffered saline with 0.1% tween (PBST), dechorionated using Protease (Sigma, P8811, St. Louis, MO, USA) treatment and fixed in 4% paraformaldehyde overnight at 4 °C. Fixed zebrafish groups were washed with PBST, followed by whole-mount staining as previously described [22,24]. We Permeabilized the embryos using PBS with 2% TritonX-100 (Sigma, X-100, St. Louis, MO, USA) before adding Phalloidin Alexa Flour 594 (red) staining (Thermofisher, A12381, 1:1000, Waltham, MA, USA) overnight at 4 °C to label actin filaments. Images of the stained zebrafish were captured using Zeiss Lumar 12 Stereomicroscope and Zeiss light-sheet microscopy. 

### 2.11. Light-Sheet Microscopy

Whole-mount immuno-stained zebrafish were imaged using light-sheet microscopy to examine brain development at 24, 72, and 96 hpf. Larvae were mounted in 1.5% low-melt agarose (Sigma, A9414, St. Louis, MO, USA) and maintained at 42 °C. Images were acquired using a W Plan-Apochromat 20× magnification/1.0 UV-VIS objective for a light-sheet microscope (Carl Zeiss Lightsheet Z.1, Munich, Germany) and processed with ZEN imaging software (version 2.3). Brain regions (including the eyes) were captured by maximum intensity projection (MIP) composites made from z-stack images.

### 2.12. Cresyl Violet Stain

Luxol fast blue staining kit from Electron Microscopy Sciences was used to perform Cresyl violet staining (Cat. 26681). The staining protocols were performed as previously described with some modifications [25]. Fixed zebrafish were washed with PBST, then permeabilized with PBS containing 2% Triton-X100 for 2 h on a rotating platform at room temperature followed by washes with PBST then hydration to 95% alcohol. The washed zebrafish were incubated overnight with Luxol Fast Blue Solution (Cat. 26681-01) at 56 °C then rinsed in 95% alcohol, followed by water. Larvae were incubated in Lithium carbonate solution (Cat. 26681-04) for 1 min then in 70% alcohol until the grey matter was clear and white matter sharply defined in the zebrafish brain. The larvae were counterstained in 0.1% Cresyl Violet Acetate, (Cat. 26681-02) dissolved in 10% Acetic Acid, (Cat. 26681-03) and) for 2 min followed by washing with 95% alcohol, then 100% alcohol. Stained larvae were mounted in 3% methylcellulose on a depression glass slide and imaged using a ZEISS microscope (Model Stemi 2000-C) with a ZEISS camera (Model Axiocam Erc 5s).

### 2.13. Histopathological Examination

The zebrafish larvae were fixed in 4% paraformaldehyde. The fixed groups were subjected to routine alcohol and xylene processing, then blocked in paraffin. Sections with a thickness of 4 μm were stained with Hematoxylin-eosin on slides. The brain was examined in samples from each group under light microscope (Nikon i200 Model) under 40× magnification.

### 2.14. Oligodendrocytes Analysis Using Tg (olig2: dsRed)

Transgenic zebrafish Tg (olig2: dsRed) were injected with MO (0.75 mM) to examine the dorsal, elongated olig2-labeled cells at 24–96 hpf. Injected embryos were screened for dsRed on a Zeiss fluorescent stereomicroscope. Larvae were then immobilized in 3% methylcellulose (Sigma, M0387) on a depression glass slide and imaged using a ZEISS stereo fluorescent microscope (Model Lumar 12) with a Nikon camera (Model DS-3). The length of olig2-labeled elongated axons at the spinal cord was measured for 6–10 axons per embryo by analyzing the acquired images using DanioScope software (version 1.1, Noldus, Wageningen, the Netherlands).

### 2.15. Zebrafish Locomotor Behavior Measurements 

#### 2.15.1. Burst Movement Analysis at 24 hpf

We assessed the tail flicking activity as previously performed [26]. Briefly, the spontaneous tail coiling was evaluated by acquiring 20 s interval video recording for the embryos at 24 hpf. The video was obtained at 60 frames per second setting using imaging source camera. Locomotion activity of embryos was evaluated by analyzing the acquired videos using DanioScope software (version 1.1, Noldus, Wageningen, Netherlands). Each embryo was selected by drawing a separate arena around its chorion to detect the movement of the tail. The locomotion activity was measured for 10–20 embryos, and the results were compared to the control groups.

#### 2.15.2. Touch-Evoked Response Assay at 72 hpf

Response to touch assay was performed to assess defects in sensory-motor function in the zebrafish model at 72 hpf, as previously described [26,27]. Larvae were placed in the middle of the petri dish (35 mm in diameter) filled with embryo medium, and a fine glass probe was used to make a gentle tactile stimulus apply to the larvae. The response was recorded for ~45 s for 1 larva at a time using a camera mounted on the Zeiss stereomicroscope (Stemi 2000). 

#### 2.15.3. Swimming Behavior Analysis at 120 hpf

Zebrafish larvae locomotor activity was monitored at 120 hpf using an automated Video-Track system (Noldus, Ethovision XT, Wageningen, the Netherlands), as described previously [28]. Larvae were placed individually in a 6-well plate in embryo medium. Larval swimming behavior was monitored in response to dark-to-light transitions (3 cycles of 10 min, each starting with the light). The Ethovision system was equipped with a BASLER camera and set to 30 frames per second. In each cycle, we recorded the frequency of movements, distance traveled, and the total duration of movements. Four technical replicates for each group were performed. The locomotor behavior was monitored, and the collected data were analyzed using Ethovision XT software. 

### 2.16. Statistical Analysis

Statistics were performed using GraphPad Prism software (GraphPad Software, version 8.0, San Diego, CA, USA). All results were presented as means of at least 3 experiments. The difference between groups was tested by one-way ANOVA followed by Dunnet test for multiple comparisons. The significance level was defined at 0.05. Significance levels are indicated in the figures as follows: * *p* < 0.05, ** *p* < 0.01, *** *p* < 0.001, **** *p* < 0.0001. 

## 3. Results

### 3.1. Clinical Findings

**Figure 1:**
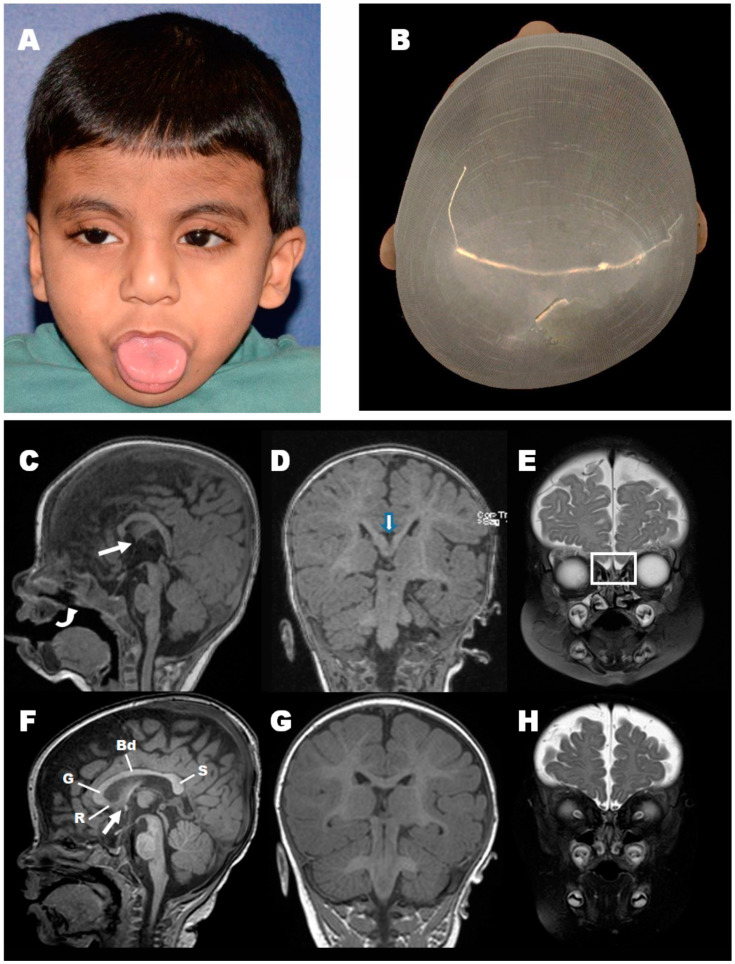
Reported case clinical presentation and Magnetic Resonance Imaging findings. Facial photos of the presented patient at 45 months of age. (**A**) Note the congenital blepharoptosis, tongue thrust posture, mild left ear constriction, mild frontal hirsutism. (**B**) Plagiocephalic head shape. (Note: The key clinical finding of cleft palate is not visible in these images). (**C**) Brain MRI showing midline sagittal T1 weighted image demonstrates a dysplastic corpus callosum with an atypical configuration showing focal thinning at the genu of the corpus callosum and an elongated vertically oriented splenium of the corpus callosum. The anterior commissure is hypoplastic (thin arrow). Cleft Palate is also noted (curved arrow). (**D**) Coronal T1 weighted image shows a cleft at the level of the splenium (thick arrow) denoting partial agenesis. (**E**) Coronal T2 weighted image shows the absence of the olfactory bulbs (Rectangle). (**F**–**H**) Similar views of an age match control show the normal configuration of the corpus callosum, which is divided into four parts: Rostum (R), genu (G), Body (Bd), and Splenium (S) as well as the anterior commissure (thin arrow). Note the olfactory bulbs on the coronal T2 weighted image (**F**).

**Figure 2:**
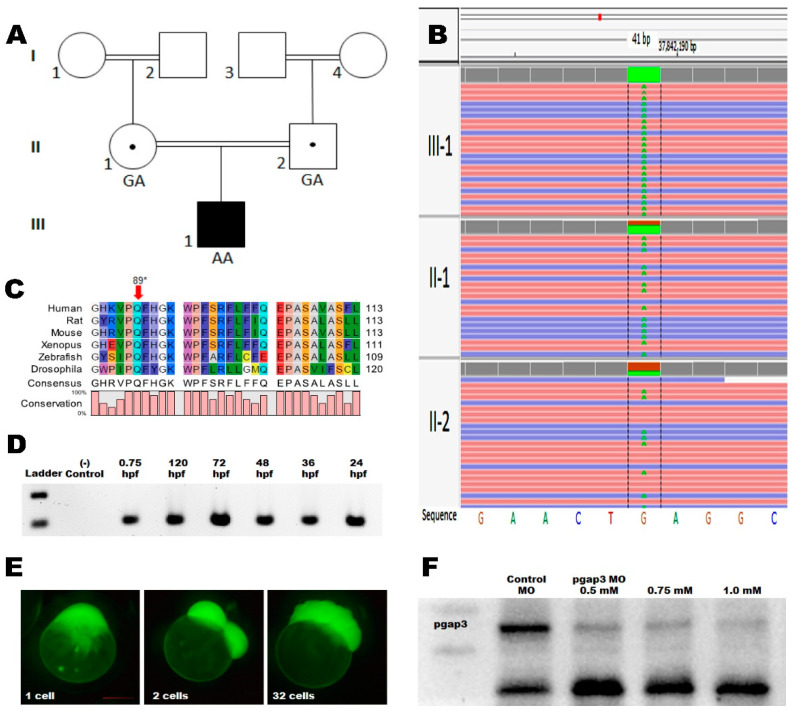
Molecular findings of the presented patient. (**A**) Pedigree of the studied family. (**B**) Sequencing gram showing *PGAP3* mutation identified by whole-genome sequencing, c.265C>T-p. Gln89*. The grandparents from both sides are 1st-degree cousins. (**C**) Multiple species alignment of PGAP3 orthologs around the affected amino acid showing highly conserved p. Gln89 residue (red arrow). PGAP3 human (NP_001278659), rat (NP_001137367), mouse (NP_001028709), xenopus (NP_001072247), zebrafish (NP_001108063), and drosophila (NP_610223.1) using CLC Sequence Viewer, Qiagen Bioinformatics. (**D**) Gene expression at different developmental stages of zebrafish development by reverse transcriptase of total RNA. (**E**) Zebrafish morpholino-FITC injections were targeting the *pgap3* ATG translation blocking site. The injected zebrafish embryo at 1-cell stage through cell divisions at two and 32-cell stage. Snap images using fluorescent imaging with Zeiss Lumar 12 microscope at 100× magnification, scale bar 200 μm. (**F**) Injected zebrafish total protein extracts analyzed by 10% SDS-PAGE, followed by immunoblot analysis using an anti-PGAP3 rabbit monoclonal antibody (1:300 dilution).

The proband was the first child for parents who self-reported as non-consanguineous, but came from a community with high consanguinity levels, suggesting a level of parental familial relationship. Of note, grandparents on both sides were 1st-degree cousins (Figure 2A). The patient was born full-term via normal delivery but was noted to have significantly reduced prenatal intrauterine movements. He was 15 months old at the time of clinical presentation and enrollment into this study. His anthropometric measurements at that time included a weight of 11.1 kg (87th percentile), a height of 92 cm (99th percentile), and an occipitofrontal circumference of 49 cm (95th percentile). Physical examination noted a cleft palate, hypotonia, stereotypy (rhythmic, repetitive hand movements), upper lid blepharoptosis with compensatory frontalis hyperactivity, occipital plagiocephaly (postural molding), intermittent habitual tongue thrusting, and mild ear asymmetry (left ear with relative conchal bowl hypertrophy) (Figure 1, Table 1). Developmental milestones were delayed and, at the latest assessment (45 months of age), reported gross motor delay and a complete lack of speech acquisition. Total blood count, serum biochemistry, metabolic screening, and thyroid functions were all normal, except for remarkably high levels of serum alkaline phosphatase (ALP), (730 IU/L, normal range 20–140 IU/L).

Neuroimaging with MRI at seven months of age showed a dysplastic corpus callosum with evidence of partial agenesis of the splenium, as well as a hypoplastic anterior commissure. The olfactory bulbs could not be visualized, denoting either olfactory bulb agenesis or hypoplasia. Myelination was mildly delayed for age (Figure 1C–E) in comparison to a healthy control (Figure 1F,G).

### 3.2. Whole-Genome Sequencing 

The patient was enrolled in the Qatari Mendelian Program, where whole-genome sequencing is offered to families to uncover genetic determinants underlying suspected monogenic disorders [31]. Using in-house pipelines (see Methods and [32], we discovered 30 rare mutations that were predicted damaging (minor allele frequency < 0.01 and CADD score > 10) and segregated with the disease. Among these, we found six novel homozygous mutations, supporting the reported degree of consanguinity. Of these novel variants, we found a homozygous nonsense variant c.265C>T (p.Gln89*) in the *PGAP3* gene (Figure 2B). The variant was completely novel vis-à-vis global databases (i.e., 1000 genomes, gnomAD, ExAC, ESP6500) and absent from 1376 controls from the Qatari population [32]. The variant was located in exon 2, suggesting an early truncation and likely complete loss of PGAP3 protein in the patient. *PGAP3* gene is associated with hyperphosphatasia with mental retardation syndrome 4. The proband presented a classical clinical presentation of HPMRS4, and was the first reported HPMRS4 case to be associated with prenatal findings of reduced intrauterine fetal movements (Table 1).

### 3.3. Zebrafish Model Has Conserved pgap3 Expression through Development

To further investigate the role of *PGAP3* in abnormal brain structures, we leveraged the zebrafish model’s unique strengths of conserved nervous system development. The zebrafish optical transparency and well-defined central nervous system make it a robust model for real-time neurological imaging and recording of motor function and behavior. The brain structures of forebrain, midbrain, and hindbrain are well defined with distinguishable boundaries at 24 h post-fertilization (hpf) [11,13]. Our analysis indicated conserved PGAP3 amino acid residue 89 among the aligned species, and that zebrafish Pgap3 protein shared 79.3% similarity with the human PGAP3 protein using CLC Sequence Viewer, Qiagen Bioinformatics (Figure 2C). In order to investigate the role of *PGAP3* in brain morphogenesis and neurodevelopment, we examined the mRNA temporal expression of zebrafish *pgap3* during the early stages of development. We saw a steady expression of *pgap3* mRNA through the different stages of development, starting from 2-cell stage to 120 h post-fertilization (hpf) (Figure 2D, Figure S1A). We also examined pgap3 expression in zebrafish embryos by whole mount in situ hybridization (WISH), the *pgap3* mRNA expression was detected at the ventricles of the developing brain at forebrain, midbrain, hindbrain and the spinal cord (Figure S1B).

To model the patient’s condition, we sought to knockdown Pgap3 protein by morpholino targeting the RNA (Figure 2E), specifically the translation site of *pgap3* transcript, and measured residual Pgap3 protein expression by western blot (see Methods). We were able to detect a dose-dependent reduction in Pgap3 protein in MO-injected groups 0.5, 0.75, and 1.0 mM doses and an overall decrease in survival rates of injected zebrafish (Figure S2A) and a negative significant impact on hatching rate (Figure S2B), for doses of 0.75 and 1.0 mM. MO injections resulted in 75.9%, 79.6%, 82.2% reduction in Pgap3 protein levels compared to normalized control protein, in a dose-dependent level (Figure 2F). 

### 3.4. Knockdown of Pgap3 Results in Neural Tube Defects in Early Zebrafish Development

**Figure 3:**
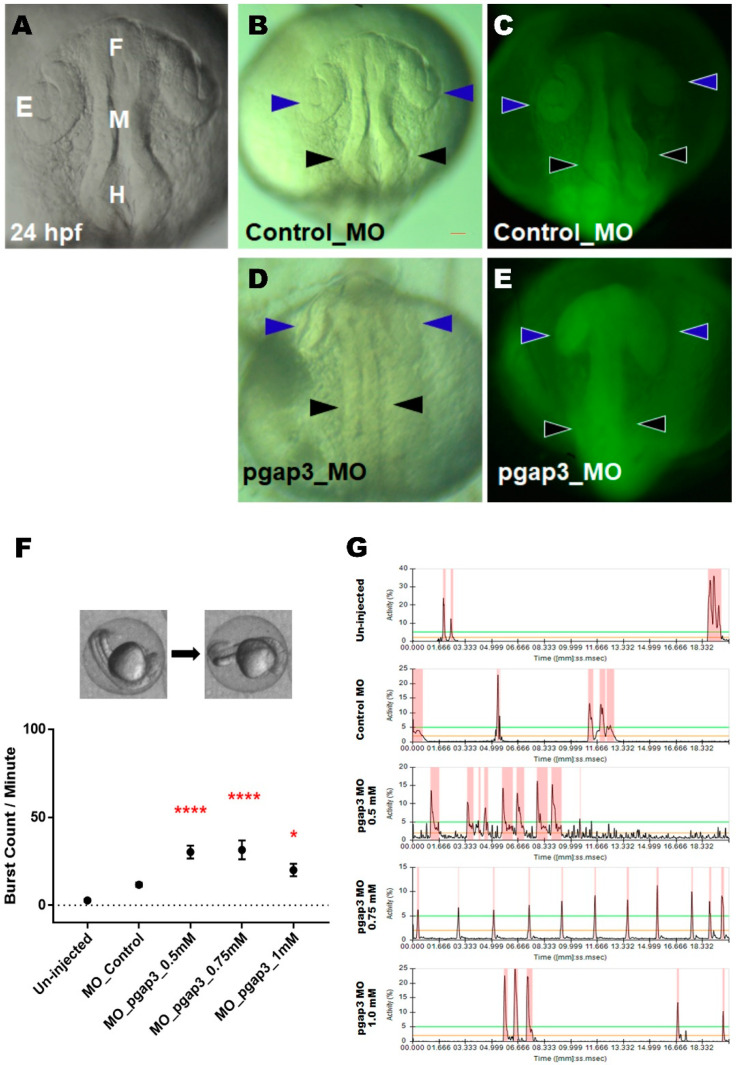
Zebrafish Pgap3 knockdown resulted in neural tube defect, delay in brain ventricles expansion, and seizure-related phenotype. Morpholino (MO)-based knockdown (0.5 mM) of zebrafish Pgap3 showed deficits in brain morphogenesis at 24 h post-fertilization (hpf) at the early stages of zebrafish brain development. Examination of *pgap3* morphants demonstrated delayed development, fused neural tube into a continuous region, reduced expansion of the brain developing parts in the anterior-posterior direction, and defects in brain ventricles formation in comparison to control MO-injected group. (**A**) Illustration of zebrafish brain development at 24 hpf demonstrating the developing zebrafish brain composed of fore, mid and hindbrain. (**B**,**C**) Zebrafish control group image at brightfield and fluorescent FITC. (**D**,**E**) Zebrafish *pgap3* morphant image at brightfield and fluorescent FITC. Arrowheads point to regions malformed in zebrafish *pgap3* morphants. E: Eye; F: Forebrain; M: Midbrain; H: Hindbrain. Zebrafish imaging using Zeiss Lumar 12 stereomicroscope with ZEISS camera (Model Axiocam Erc 5s) at a magnification of 100×, scale bar 10 μm. (**F**) Zebrafish *pagp3* morphants displayed significantly increased spontaneous head and tail coils at 24 hpf in comparison to the control group (un-injected); * *p* < 0.05, **** *p* < 0.0001. Locomotive behavior of zebrafish embryos after *pgap3* MO injections assessed by spontaneous tail flicking and embryos movements that were measured by embryo burst count per minute and plotted into column graph for the mean with the standard error of the mean. N = 25, 34, 51, 36, 38 for the Control (un-injected), Control MO, *pgap3* MO 0.5, 0.75, 1.0 mM, respectively. Statistical analysis was performed with one-way ANOVA followed by Dunnett’s test for multiple comparisons using GraphPad Prism (version 8.0). (**G**) Representative activity chart of zebrafish embryo at 24 hpf measuring movements through time for the zebrafish examined groups. Zebrafish imaging using Zeiss Lumar 12 stereomicroscope at a magnification of 10× supplied with Imaging source camera DMK 33UX252.

Brain morphogenesis within zebrafish begins after neural tube closure at 17 hpf [33]. The zebrafish formation of the brain ventricles will delineate central nervous system functional units and will be defined into forebrain, midbrain, and hindbrain (Figure 3A) [33,34]. The zebrafish embryos were screened and selected based on ubiquitous FITC expression (Figure 2E). MO-based knockdown of zebrafish Pgap3 resulted in embryonic disruptions of brain morphogenesis at the early stages of zebrafish development in comparison to the control group (Figure 3B–E). Gross morphological examination at 24 hpf of *pgap3* morphants demonstrated delayed development, a fused neural tube, reduced expansion of the brain developing parts in the anterior-posterior direction, and defects in defined brain ventricles formation (Figure 3D,E) in comparison to control MO-injected group (Figure 3B,C).

### 3.5. Zebrafish Pgap3 Knockdown Altered Neuronal Wiring at Early Stages of Development

During the early stages of development, zebrafish have a strong locomotive behavior that reflects the neural networks in the developing brain and spinal cord. Spontaneous tail coiling (due to innervation of the muscle by primary motoneurons) is the first motor activity generated by the developing simple spinal network [26,35,36,37,38]. This swimming ability through the different zebrafish developmental stages is critical and provides links to the normal development of the brain and spinal cord. To examine the zebrafish morphants’ locomotive behavior, spontaneous tail coiling, and embryos contractions were evaluated at 24 hpf (Supplementary material Videos S1–S5). Under normal conditions, zebrafish embryos show a burst of 3–5 coils, often followed by a period of ~20 s of inactivity [26]. *Pgap3* knockdown resulted in a significant increase in spontaneous coils of zebrafish embryos at 24 hpf in comparison to the control group. Zebrafish morphants displayed a mean of 30, 32, and 20 bursts per minute for the 0.5, 0.75, and 1.0 mM MO doses (Figure 3F,G), respectively, compared to 11 and 3 bursts per minute for the control MO and un-injected groups (Figure 3F,G, Video S1). 

Our results indicated that *Pgap3* loss resulted in altered neuronal wiring of the zebrafish developing brain and spinal cord, leading to an increase in spontaneous movements that resembled twitching and hyperactivity that are related to epileptic-like movements. However, these movements did not affect the hatching rate in these *pgap3* groups. Our results indicated that Pgap3 KD resulted in reduced hatching rate at 54%, 38%, and 41% for the *pgap3* MO-injected doses of 0.5, 0.75, and 1.0 mM, respectively, when compared to a control group rate of 87% (Figure S2B). The ratio of hatching embryos is a well-established staging index for the developmental stage of the zebrafish model [38] and similar delays in zebrafish hatching rate were previously reported to be associated with developmental delays and levels of neurotransmitters [39], suggesting an underlying neurological abnormality. 

### 3.6. Zebrafish pgap3 Morphants Display Dysmorphic Features Resembling Human HPMRS4

**Figure 4:**
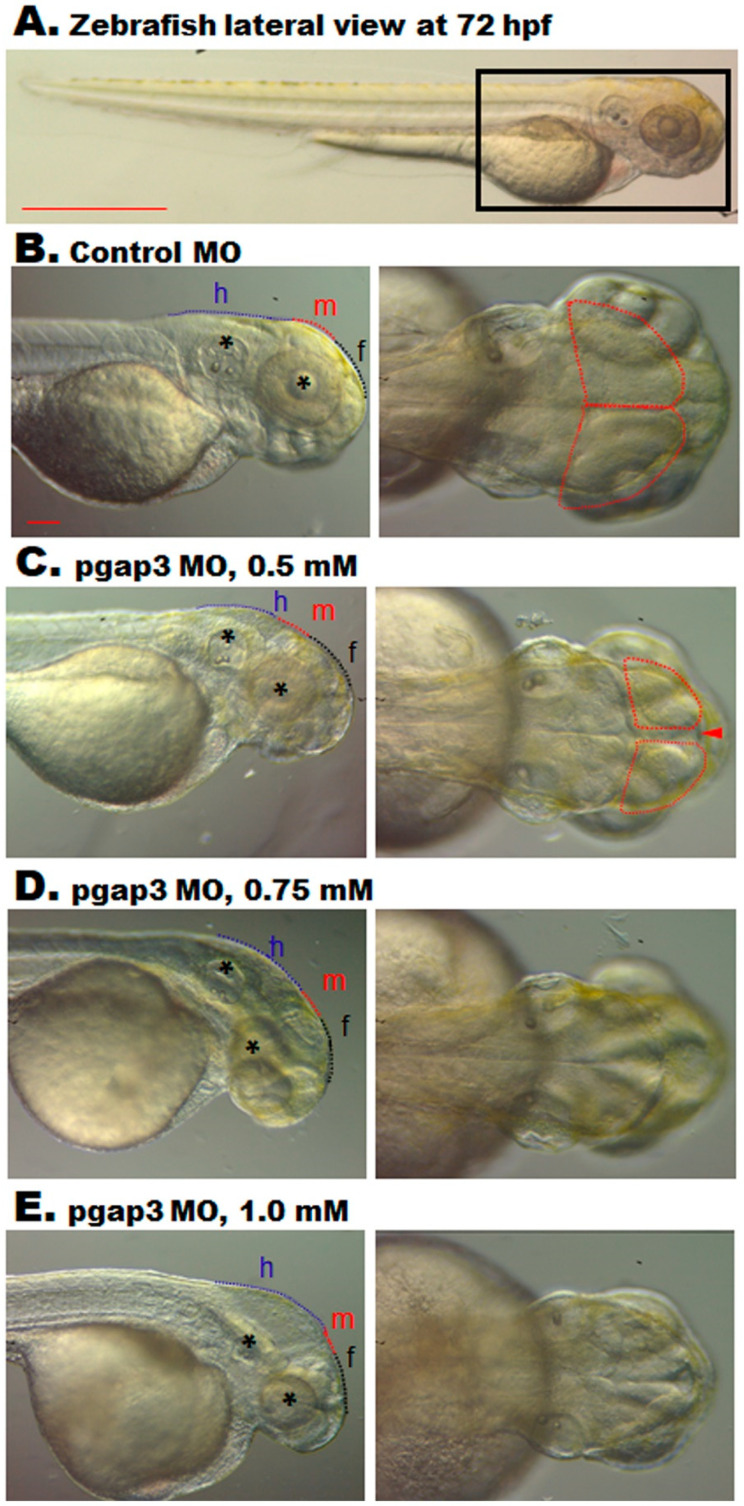
Knockdown of Pgap3 resulted in zebrafish developmental deformities. (**A**) Control zebrafish full-body positioned at the lateral view at 72 h post-fertilization (hpf), black box zooming on the zebrafish head at a magnification of 32×, scale bar 100 μm. (**B**) Gross examination of the control group at a lateral and dorsal view of the zebrafish head showing normal brain development, normal forebrain, midbrain that typically expanded to the left and right tectal ventricles and hindbrain. Normal head orientation demonstrated by a black asterisk indicating the position of the ear and eye. (**C**–**E**) The knockdown of Pgap3 resulted in developmental brain deformities. Examined zebrafish pagp3 morphants showed brain defects and dysmorphic facial features that demonstrate a dose-dependent severity. Zebrafish *pgap3* morphants displayed a delay in brain development, separation (red arrowhead) between the left and right ventricles of the midbrain ((**C**) traced by dotted red shape), and reduction in head size relative to control and low sets of ears in comparison to eye position (black asterisks). Dotted black, red, and blue lines illustrate the forebrain (f), midbrain (m), and hindbrain (h) structures, respectively. Experiments performed three times, and the total number of embryos examined: 30, 40, 45, 35 for control MO, *pgap3* MO 0.5, 0.75, 1.0 mM, respectively. Zebrafish imaging using Zeiss Lumar 12 stereomicroscope with a ZEISS camera (Model Axiocam Erc 5s) at a magnification of 100× for head images, scale bar 10 μm.

Phenotypic analysis by gross morphological examination demonstrated that Pgap3 knockdown resulted in developmental head deformities. Zebrafish *pgap3* morphants displayed a reduction in the cephalic region and dysmorphic head features showing a bent head and low set of ears at 72 hpf (Figure 4). Head orientation abnormalities were initially observed in *pgap3* morphants and associated with a deficit in brain ventricles expansion, midbrain conformation, and midbrain–hindbrain boundary (Figure 4C–E). In comparison to the control group (Figure 4A,B), the zebrafish showed a dose-dependent hypoplastic midbrain size, alteration in midbrain-hindbrain boundary, and significant delay in brain development (Figure 4C–E). Furthermore, the larval phenotypes mimic the dysmorphic features and brain anomalies exhibited by individuals with pathogenic variants in *PGAP3.* These characteristics get progressively worse with higher morpholino doses, suggesting that they result from depletion of Pgap3 protein in the developing zebrafish, demonstrating in vivo the essential function of this protein during the early stages of development.

### 3.7. Loss of Pgap3 Resulted in Zebrafish Midbrain Tectum and Cerebellum Defects

**Figure 5:**
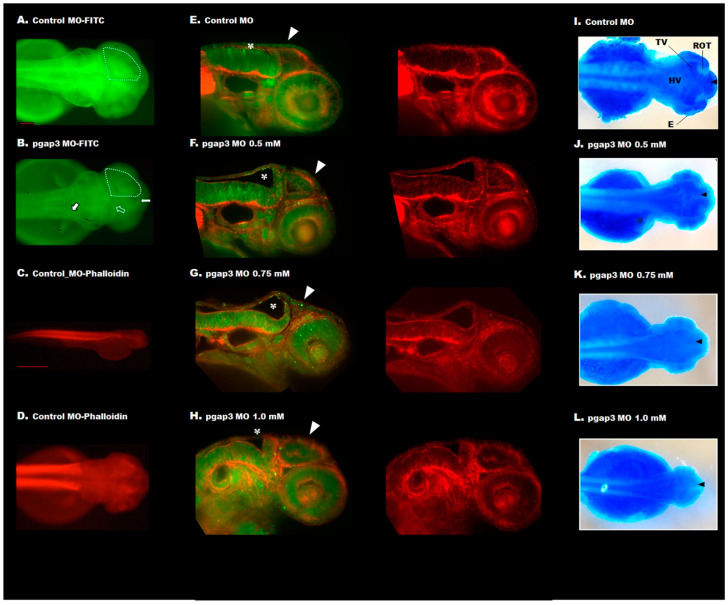
Knockdown of Pgap3 resulted in zebrafish in dose-dependent midbrain tectum and cerebellum defects. (**A**) Zebrafish injected with control MO-FITC displayed normal brain development, normal midbrain with expanded left and right tectal ventricles at 72 h post-fertilization (hpf). (**B**) Zebrafish injected with *pgap3* MO-FITC showed failure for developing tectum that connects the left and right ventricles of the midbrain (traced by dotted white shape) demonstrated by clear separation (white arrow), impaired midbrain-hindbrain boundary (empty white arrow), and brain stem malformation (black-white arrow). (**C**,**D**) Whole-mount immunofluorescence zebrafish using phalloidin to stain actin filaments (red). Zebrafish imaging using Zeiss Lumar 12 stereomicroscope, full-body at 32×, scale bar 100 μm, and dorsal view at 100× magnification, scale bar 10 μm. (**E**–**H**) Examination of the zebrafish midbrain using light-sheet imaging for two channels of red (actin) and green (MO-FITC), images of red channel (phalloidin stain of actin) is included. (**E**) Control zebrafish had normal brain development, normal midbrain with connected. They expanded left and right tectal ventricles, white arrowhead showing the tectum, and white asterisk showing the position of the cerebellum. (**F**–**H**) The knockdown of Pgap3 resulted in developmental brain deformities. Examined zebrafish *pgap3* morphants showed brain defects and widely separated ventricles. Imaging using light-sheet microscopy at magnification 16×. (**I**–**L**) Separation of the tectal zebrafish left and right ventricles demonstrated by Luxol blue-cresyl violet staining. Anatomy of the zebrafish brain: E, eye; midbrain parts: OT, optic tectum (R, right); TV, tectal ventricle and HV, hindbrain ventricle. Zebrafish imaging using Zeiss Lumar 12 stereomicroscope with a ZEISS camera (Model Axiocam Erc 5s) at a magnification of 32×.

**Figure 6:**
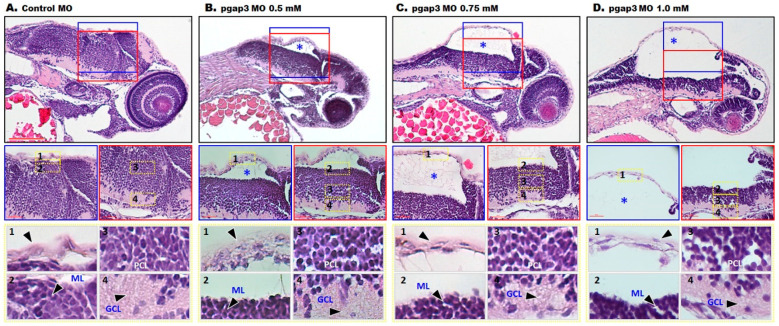
Histopathology of zebrafish Pgap3 knockdown model. Images of Hematoxylin and Eosin stained brain tissue sagittal sections from zebrafish larvae at 72 h post-fertilization (hpf). (**A**) Zebrafish injected with control MO displayed normal brain development, normal brain epithelium with an expanded molecular layer (ML), Purkinje cell layer (PCL), and granule cell layer (GCL). Subsets of the examined brain sections, showing the four components: 1: Epithelium, 2: ML, 3: PCL, 4: GCL. (**B**–**D**) Zebrafish injected with *pgap3* MO revealed brain anomalies at the cellular level demonstrated by the increased separation between the brain ventricles (blue asterisk), and a reduced area size and cellular density at the three layers of the developing brain (black arrowheads showing the cellular components of the examined layer). Images captured using Nikon i200 Model under 10×, scale bar: 200 μm and 40× magnification, scale bar: 40 μm.

Consistent with the clinical findings of corpus callosum agenesis in HPMRS4 patients, zebrafish *pgap3* morphants displayed failure of developing a normal tectum that connects the left and right ventricles of the midbrain, a vital processing center for sensory information, by 72 hpf (Figure 4 and Figure 5). The morphants displayed clear separation of the two ventricles (Figure 4C–E, Figure 5B,F–H,J–L), impaired midbrain–hindbrain boundary formation and brain stem malformation in comparison to control zebrafish that had normal brain development, normal midbrain with connected and expanded left and right tectal ventricles and normal cerebellum formation (Figure 4A,B, Figure 5A,E,I). These findings were further supported by light-sheet imaging (Figure 5E–H) and luxol blue -cresyl violet staining (Figure 5I–L). Luxol fast blue stain is used to identify myelin in nervous tissue. The myelin proteolipid will display a dark blue precipitate, the gray matter will be colorless, and the white matter will be blue [25]. In contrast, to the control group (Figure 5I), *pgap3* morphants displayed distanced tectal ventricles and impaired myelination of the nervous tissue was observed under Luxol fast blue staining (Figure 5J–L) mimicking hypomyelination seen in some HPMRS4 patients. The histopathological examination revealed a prominent difference in terms of degenerative changes in brain tissue of the zebrafish Pgap3 knockdown model. The model showed reduced brain size, dilated ventricle, poorly organized brain cellular components (Figure 6B–D). The examined brain three layers (molecular layer, Purkinje cell layer, and granule cell layer) established the effect of Pgap3 on the developing brain showing a dose-dependent reduced area size and cellular density (Figure 6B–D, blue and red square) with degeneration of the granule layer (Figure 6B–D, subsets 4) of the cerebellum that comprise the largest population of neurons in the vertebrate central nervous system (Figure 6). 

### 3.8. Impaired Sensory Response in Zebrafish pgap3 Morphants 

**Figure 7:**
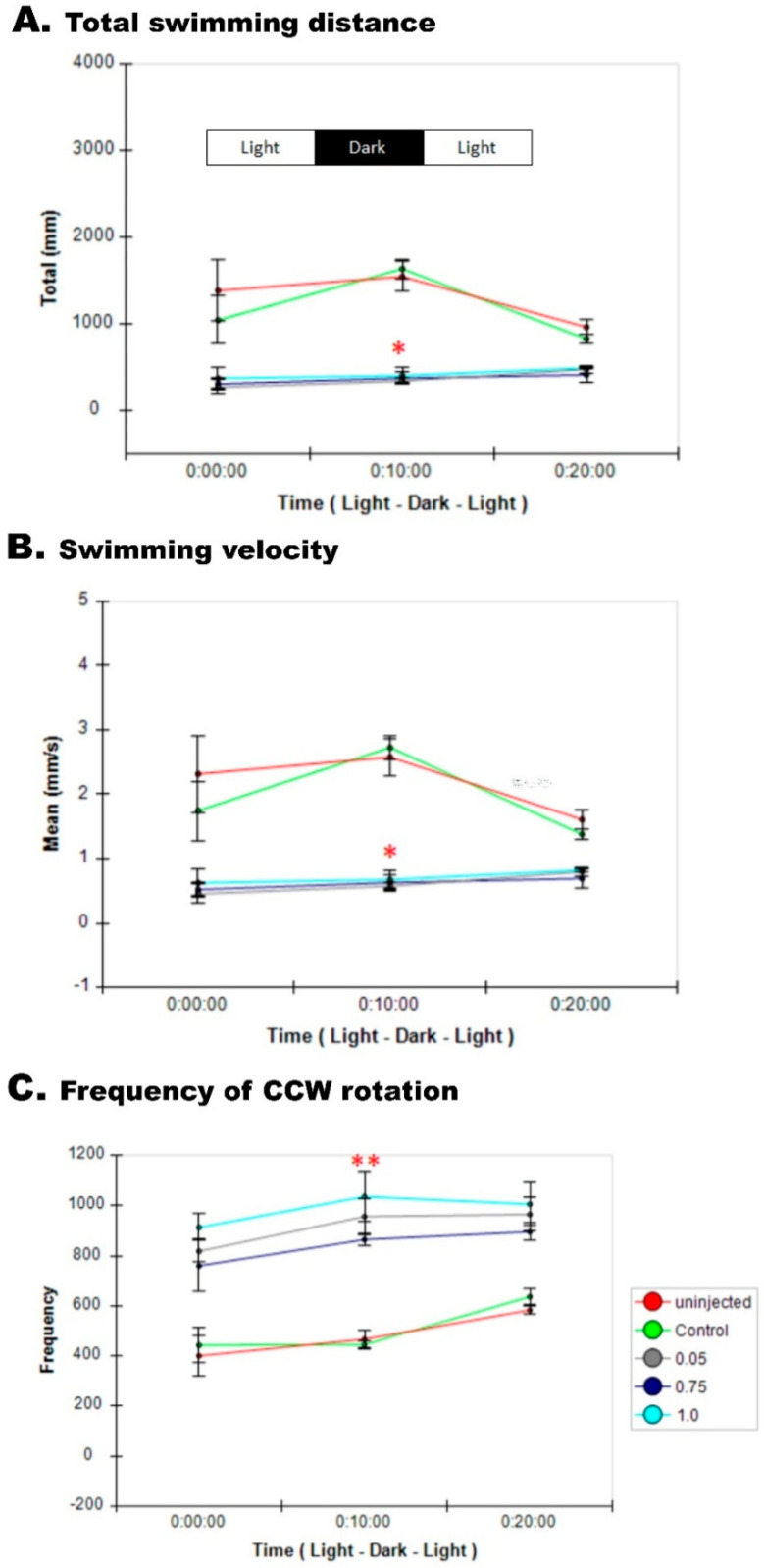
Impaired sensory response in zebrafish *pgap3* morphants. Knockdown of Pgap3 resulted in reduced responses to the sensory trigger represented by less velocity and total distance moved per larvae over time assessed by locomotive behavior recording through the presentation of light-dark cycles over a time interval of 30 min. (**A**) Total distance moved by larvae calculated using Ethovision software (Noldus), *pgap3* morphants had significantly less total distance moved, * *p* < 0.05. (**B**) Velocity of larvae calculated using Ethovision software (Noldus), *pgap3* morphants had a significant reduction in velocity, * *p* < 0.05. (**C**) Frequency pf counter-clockwise rotation of larvae calculated using Ethovision software (Noldus), *pgap3* morphants had a substantial increase in frequency, ** *p* < 0.001. The total number of larvae examined: 6 from each group of uninjected, control MO, *pgap3* MO 0.5, 0.75 and 1.0 mM. Statistical analysis was performed with one-way ANOVA followed by Dunnett’s test for multiple comparisons using GraphPad Prism (version 7.0).

The zebrafish tectum connections structure in the midbrain represents the brain circuit for the transformation of sensory input into movement output that its mammalian counterpart is the superior colliculus structure [11]. This process involves neural sensory responses through a network of tectal neurons and circuits to induce motion. We assessed sensorimotor action in larval zebrafish at 120 hpf by the presentation of light-dark cycles over the time interval of 30 min and recorded locomotive behavior. Knockdown of Pgap3 resulted in significantly reduced responses to the sensory trigger of dark-light cycle exposures represented by the less total distance moved per larvae over time (Figure 7A). 

### 3.9. Zebrafish pgap3 Morphants Display Impaired Touch Sensitivity and Seizure-Related Phenotype

To study the neuromuscular function of the developing zebrafish, at 72 hpf zebrafish larvae were subjected to a poking test, in which the embryo was tapped with an embryo poker, and the response movement recorded using real-time video capturing (see Methods). The swimming response was impaired in comparison to the control, as expected, *pgap3* morphant showed a dose-dependent loss of touch response. While with continued touching, these morphants became desensitized to the stimulus (Video S2B–D). The observed abnormal motor behavior included a spastic movement with a seizure-like swimming response in comparison to the control fish (Video S2A). 

The seizure-like behavior involved involuntary, rapid movements of the body observed in larvae zebrafish at 120 hpf with spiral swimming, circling, spasms, and tremor locomotion as quantified by assessing the velocity and distance traveled and spiral rotation count by automated video-tracking tools (see Methods). The total moved distance and velocity were significantly decreased in the *pgap3* morphants compared to control groups (n = 6 for each examined group). The mean total distance for the control groups ranged between 1200–1300 millimeters (mm) with a mean velocity of 2.0–2.2 mm per second (mm/s) (Figure 7A,B). While for the morphants, a total distance of 390–420 mm with a velocity of 0.65–0.7 mm/s (Figure 7A,B). In contrast, the frequency of the counter-clockwise (CCW) rotation was higher in *pgap3* morphants capturing an increase in the spiral and circling moves of these morphants. The average frequency for CCW was around 510–530 for the control groups, while it ranged from 900–1000 rotations in *pgap3* morphants (Figure 7C). These observations of spiral and circling moves were previously described as endpoints of seizures in zebrafish embryos [13,36,40]. Together, our findings of early epileptic-like movements during the early stage of development at 24 hpf (Figure 3F,G, Video S1C–E), followed by impaired sensory response and loss of touch at 72 hpf (Video S2B–D) then reduced response and movement at 120 hpf (Figure 7) that associated with less distance, slower velocity, and increased frequency of counter-clock rotations of the swimming sensory response in *pgap3* morphants, resembled the human patient’s clinical phenotype.

### 3.10. Loss of PGAP3 Function Impaired Oligodendrocytes Expression Resulting in Shorter Motor Neuron Axons

We suspected that the defects observed at early stages of zebrafish development, including altered locomotor behavior, were a result of a disorder in neuronal wiring in the zebrafish *pgap3* morphant’s developing brain and spinal cord, explaining the etiology of hypotonia, delayed myelination and seizure phenotypes, which are common among HPMRS4 patients [3]. In larval zebrafish, the developing retinal ganglion cell axons reach the tectum by about 44 h, close to when the photoreceptor layer finishes development, and begin forming arbors around 60 hpf (Stuermer, 1988). Shortly after this, at 72 hpf, the first visually evoked responses are seen in the fish (Easter and Nicola, 1996). 

**Figure 8:**
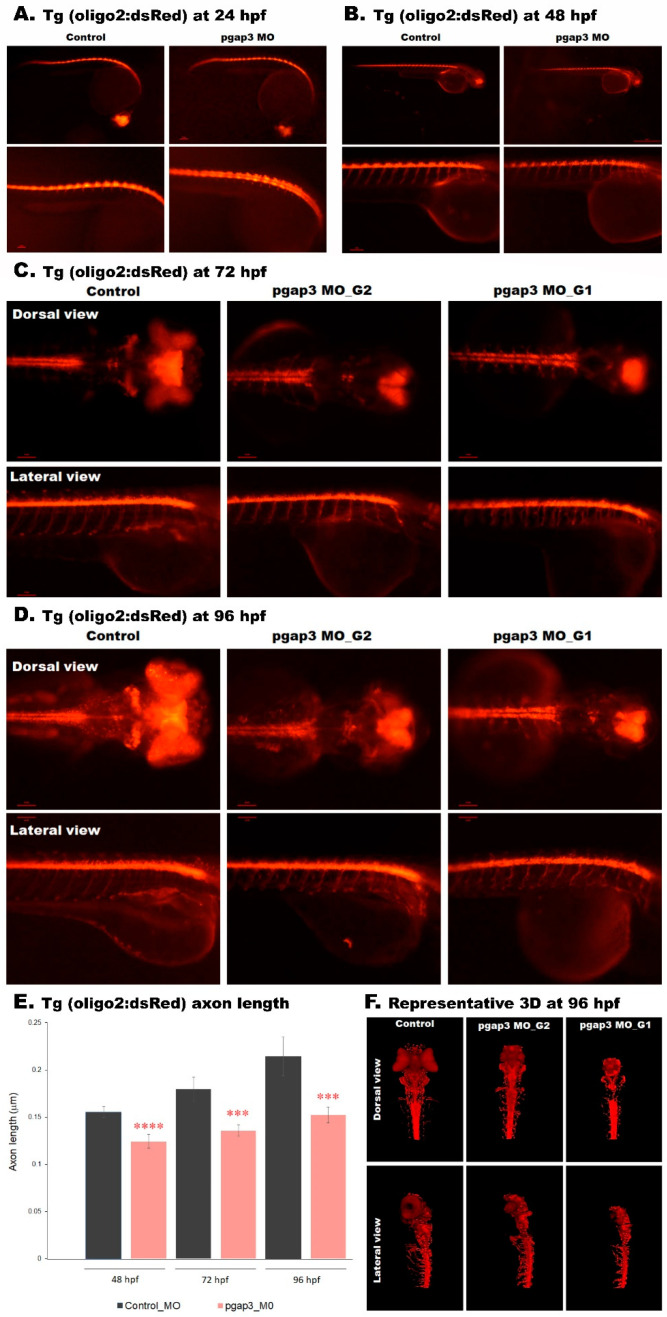
*pgap3* morphants displayed reduced oligodendrocytes (OPCs) expression and shorter motor axons. Transgenic embryos (Tg: olig2: dsRed, expressing red fluorescent oligodendrocytes (OPCs) and motor neurons axons) were injected with control and *pgap3* morpholino (0.75 mM). Representative embryos full-body lateral view and trunk lateral view at (**A**) 24 h post-fertilization (hpf) whole body at magnification 63×, scale bar 10 μm and lateral view at magnification 100×, scale bar 10 μm. (**B**) 48 hpf full body at magnification 32× and lateral view at magnification 100×. (**C**) Morphant zebrafish MO_G2 (moderate phenotype) and MO_G1 (severe phenotype) at 72 hpf. (**D**) Morphant zebrafish MO_G2 (moderate phenotype) and MO_G1 (severe phenotype) at 96 hpf. Dorsal and lateral view at a magnification of 100×. (**E**) Axon length measured for 8–10 axons per embryo, *pgap3* morphants (*n* = 10) showed significantly shortened axons compared to controls (*n* = 9), at 48 hpf, **** *p* < 0.0001; 72 hpf, *** *p* < 0.001; and 96 hpf, *** *p* < 0.001. (**F**) *pgap3* morphants displayed a reduction in OPCs of the developing brain and optic area compared to controls at 96 hpf. Dorsal view of a representative embryo showing OPCs labeled with cytoplasmic dsRed. Lateral view demonstrated shorter motor axons at the trunk examined region of the zebrafish compared to the olig2-labeled elongated cells in the control group. One-way ANOVA with Dunnett’s multiple comparison test was used for statistical analysis. Zebrafish imaging using Zeiss light-sheet microscopy at a magnification of 8×. G1 sever phenotype, G2 moderate phenotype.

We sought to investigate the Pgap3 knockdown effect on the developing zebrafish motor neurons. It is known that in the developing zebrafish spinal cord, motoneurons originate from oligodendrocytes that are derived from a brain ventral region [41]. We examined the Pgap3 KD model (0.75 mM) in the Tg (olig2:dsRed) line in time course through zebrafish development, where the *olig2* gene regulatory sequence drives expression of dsRed in motor neurons, interneurons, and oligodendrocyte precursor cells (OPCs). At 24 and 48 hpf, we observed *olig2+* cells present along the spinal cord with subtle differences between the examined groups (Figure 8A,B). However, at 72 and 96 hpf, we observed significantly fewer olig2-expressing cells in the forebrain, midbrain, and hindbrain, and the shorter and less extended axons were seen at the spinal cord of the Pgap3 Knockdown compared to the control group (Figure 8C–F). 

Examination of control olig2-expressing zebrafish showed widely oligo2 enriched areas around the cerebellum at 72 hpf and 96 hpf stage. Whereas, the examination of olig2-expression in the morphant zebrafish showed MO_G2 (moderate phenotype) and MO_G1 (severe phenotype) that OPCs were restricted to the cluster and stripe of the cerebellum and almost absent at the cerebellum (8D). To assess whether the abnormal expression of oligodendrocytes will result in any defects in myelination in the pagp3 KD model, we examined the myelinated axons in the ventral spinal cord. The distribution of myelinated axons in the *pgap3* morphant was significantly impaired per examined segment of the ventral spinal cord compared to the control group (Figure 8C,D, Video S3B,C). Measurements of axon length demonstrated significantly shorter axons in the *pgap3* morphant group (Figure 8E,F). The average axon length per embryo was 0.125 μm compared to 0.151 μm at 48 hpf, 0.134 μm compared to 0.178 μm at 72 hpf, 0.154 μm compared to 0.214 μm at 96 hpf for morphants (0.75 mM and 1.0 mM) and control groups, respectively. These results demonstrate the importance of Pgap3 in oligodendrocytes migration, distribution, and myelination in early stages of development, features that are challenging to observe in human development.

## 4. Discussion

In this study, we present the unique case of a 3-year old boy with dysmorphic facial features, global developmental delay, cleft palate, and neuromuscular abnormalities. The subject was enrolled in the Qatari Mendelian Program–a multi-stakeholder effort providing whole-genome sequencing to solve rare idiopathic disorders in the Qatari population. Using a combination of in-house and best-practices pipelines, we discovered a novel pathogenic *PGAP3* nonsense mutation, p.Gln89*, supporting a diagnosis of HPMRS4. Brain neuroimaging uncovered corpus callosum dysplasia with apparent partial agenesis of the splenium as well as a hypoplastic anterior commissure. The olfactory bulbs were not visualized denoting olfactory bulb agenesis or hypoplasia. Additional blood investigations revealed increased alkaline phosphatase levels, which further support a diagnosis of HPMRS4. Importantly, this patient is the first reported HPMRS4 to be associated with prenatal findings of reduced intrauterine fetal movements. To date, only 18 pathogenic *PGAP3* mutations have been reported in HPMRS4 patients associated with brain anomalies, all with unremarkable prenatal history [3]. Thus far, all reported HPMRS4 patients presented with hypotonia, developmental delay, and intellectual disability, while >50% of these patients have seizures and brain malformations. These reported brain anomalies include thin corpus callosum, dilated ventricles [1], cerebellar vermis hypoplasia, cortical dysplasia, and Dandy–Walker malformation [42], cerebellar vermis hypoplasia, and a mild ventriculomegaly [5], brain atrophy of the temporal lobes, mega cisterna magna, and small capsula interna [43]. More anomalies were as well reported by Pagnementa et al. [44] in a family with *PGAP3* mutations—MRI detected a mild variant of Dandy–Walker malformation. At the same time, the brother’s MRI showed a mild generalized lack of white matter bulk and small olfactory bulbs. Additionally, a recent report showed frontoparietal atrophy with bilateral enlargement of lateral ventricles in one subject and callosal dysgenesis with a hippocampal minor structural anomaly in the other subject [30].

Nevertheless, a recent report [3,42] indicated that more cases are required to confirm the association between brain anomalies and HPMRS4.

Our novel mutation, together with the prenatal presentation, expands the reported phenotypic and genotypic spectrum of this rare recessive disorder and suggest the origins of this disease early in neuronal development. We sought to elucidate the developmental origins of this disorder using the zebrafish model. 

Using targeted knockdown of Pgap3 in zebrafish, we observe neural tube defects, delayed brain ventricle expansion, widening between brain ventricles, altered neuronal wiring, and phenotypes consistent with developmental delay. Moreover, gross morphological abnormalities of the morphant were noticed, including dysmorphic cranial and facial features and low set position of ears. These observed phenotypes mimic the clinical hallmarks and developmental defects seen in HPMRS4 patients, suggesting a novel and essential role of *PGAP3* at the early stages of brain development and morphogenesis. 

The zebrafish neural tube defects observed, a phenotype that has not been documented in humans who are ascertained later in childhood, suggests that structural brain anomalies in HPMRS4 cases may arise during fetal brain formation as early as the third week of gestation [14]. 

Given the role of brain structure in shaping the fetal skull formation and facial features [45]; the HRMSR4 facial dysmorphia may be a putative consequence of an early fetal brain morphogenesis defects. 

In addition to explaining HRMSR4 physical features, our zebrafish model enabled the dissection of neurological impairment related to HRMSR4. For example, we showed for the first time that reduction in Pgap3 protein levels could lead to a reduced number of oligodendrocytes within the ventricular zone of the developing brain (Figure 8) and reduced motor neuron axon length (Figure 8E), resulting in an altered sensory response, unusual touch sensitivity, and a seizure-like phenotype. 

The human oligodendrocytes originate in the brain subventricular zone then migrate to populate the developing white and grey matter and spinal cord to differentiate into myelinating cells and motor neurons. The first synapses begin forming in a fetus’s spinal cord at 5 weeks of gestation, and by 6–7 weeks, the first fetal movements of spontaneous motoneuron activity will take place [46,47]. Consequently, any alteration or defects in this process can impair the nervous system function. 

Our findings establish that *PGAP3* is essential for neural oligodendrocytes formation. The morphants displayed increased burst movement at the early stages of development, indicating increased release of neurotransmitters. Further, a recent study has established via simultaneous measurements of brain optical neuronal activity and locomotor behavior of the zebrafish larvae that increased neuronal bursts represent epileptic seizures [48,49]. It has been posited that excessive release of neurotransmitters may be damaging to oligodendrocytes and result in impairment of neurological function [50]. These morphants at later stages of development expressed fewer oligodendrocytes, decreased myelination at the brain tectal ventricles, and shorter axons at the ventral spinal cord. Altogether, these abnormalities lead to a weakened induction of a motor response. Our results point to an early role of *PGAP3* during fetal motoneuron development, explaining the remarkable prenatal reduced fetal movements in our patient. 

Additionally, as HPMRS4 patients develop seizures and hypotonia; this may be due to motor neuron dysfunction that ought to take place in a developing fetus. Further, suggesting a possible therapeutic opportunity for halting the disease progression by developing interventions aimed at maintaining axonal survival and promoting remyelination. 

Altogether, the present study identified a novel recessive *PGAP3* mutation associated with a unique clinical presentation of HPMRS4 in a patient with south Asian ancestry. By leveraging the throughput, transparency, and genetic manipulation of zebrafish, we uncover an essential role for *PGAP3* at the earliest stages of neurological development. 

Further, through serial knockdowns, we showed a dose-dependent effect in producing brain defects, which may explain the variability in clinical symptoms based on residual gene function in HPMRS4 patients and explain the severity in our patients with a homozygous nonsense mutation. Finally, the aberrant motor neurons development in our model points to the possibility of early intervention in at-risk families by employing therapies that improve neuronal growth and differentiation at early stages development to overcome the underlying genetic defect in such patients. 

## 5. Conclusions

In this study, we report a HPMRS4 patient with a novel nonsense mutation in *PGAP3* exhibiting a unique clinical presentation of noticeably reduced fetal intrauterine movements associated with dysgenesis of the corpus callosum and olfactory bulb agenesis. To validate the *PGAP3* role in brain morphogenesis, we generated and characterized a Pgap3 KD zebrafish model using functional assessment and behavioral analysis. Zebrafish morphants displayed impairments mimicking the human phenotypes of HPMRS4. These results indicate a functional deficiency of PGAP3, leading to brain anomaly in HPMRS4 patients and revealing an essential role of *PGAP3* in neural tube development and expansion. Our model revealed a direct correlation of Pgap3 loss and oligodendrocytes abundance and motor neuron development, leading to impairments in sensory and locomotor behavior. Importantly, elucidating the role of *PGAP3* in brain development may provide valuable insights into the etiology of these rare disorders that can be exploited further in future studies to screen for such anomalies through fetal screening during pregnancy. 

## Highlights

- This study of a novel *PGAP3* variant represents the first HPMRS4 case reported with unique clinical features of decreased fetal intrauterine movements and olfactory bulb agenesis
- Functional modeling of the PGAP3 deficit revealed the impact on neural tube development and expansion at early stages of zebrafish development
- *PGAP3* plays a novel role in brain morphogenesis
- *PGAP3* is essential for neuronal oligodendrocytes migration, distribution, and myelination in early stages of development
- PGAP3 Knockdown resulted in motor neuron deficits, sensory, and locomotor impairments

## Figures and Tables

**Table 1:** Clinical features of the study proband and previously reported cases with *PGAP3* frameshift or protein-truncating mutations. Abbreviations: DD, developmental delay; ID, intellectual disability; GU, genitourinary; GI, gastrointestinal; GERD, gastroesophageal reflux disease; MRI, magnetic resonance imaging; CT, computed tomography.

Clinical Feature	Proband II-1	Previously Reported Cases with Loss-Off-Function Variants				
	This Study	Abi Farraj et al. 2019 [29]	Abdel-Hamid et al. 2018 [5]	Howard et al. 2014 [1]	Dogan et al. 2019 [30]	Abdel-Hamid et al. 2018 [5]
	p.Gln89*	p.C68LfsX88	p.M135Hfs*28	p.Leu147Profs*16, p.Asp305Gly	p.Tyr169*	p.D273Sfs*37
**DD, developmental delay; ID, intellectual disability**	yes	yes	yes	yes	yes	yes
**Neurological abnormalities (seizures, hypotonia)**	yes, only hypotonia	yes	yes	yes	yes, only hypotonia	yes
**Dysmorphic facial features**	yes, cleft palate	yes, cleft palate	yes	yes	yes, cleft palate	yes
**Cranial shape anomalies**	yes, plagiocephaly	no	no	no	yes, postnatal microcephaly	no
**Deafness**	no	no	yes, hearing loss	N/A	no	no
**Ophthalmological anomalies**	yes, poor vision	no	yes, optic nerve pallor	N/A	no	no
**Cardiac anomalies**	no	yes, Congenital Heart Defect (CHD)	yes, patent ductus arteriosus (PDA) and atrial septal defect (ASD)	N/A	yes, left ventricle hypertrophy	no
**GU, genitourinary malformation**	no	yes,	yes, undescended testicles	N/A	no	yes, hypoplastic clitoris, absent labia minora
**GI, gastrointestinal anomalies including GERD gastroesophageal reflux disease**	yes, swallowing dysfunction	yes, esophagitis, hiatal hernia, petechial gastritis, nodular duodenitis	yes, inguinal hernia, hepatosplenomegaly	N/A	yes, dysphagia, umbilical hernia	no
**Nephrocalcinosis**	no	N/A	no	N/A	no	no
**Teeth anomalies**	no	N/A	yes, double row teeth	N/A	no	no
**Nail anomalies**	no	no	no	N/A	yes, brittle hypoplastic nails	no
**Short fingers or hands**	no	no	no	no	no	no
**Hand/feet anomalies**	Yes, hands clawing, feet talipes deformity	yes, clinodactyly of 5th digit	no	no	no	no
**Skeletal findings**	yes	no	yes	no	no	yes, pectus excavatum
**Brain MRI/CT anomalies**	yes, thin corpus callosum	yes, left temporal lobe bleeding	yes, thin corpus callosum	N/A	yes, thin corpus callosum	no
**Prenatal finding**	yes, decreased IUM	no	N/A	N/A	no	N/A
**Elevated serum alkaline phosphatase**	yes	yes	yes	yes	yes	yes
**other**	tongue thrust posture, mild left ear constriction	skin hyperpigmentation	squint, protruding tongue, nystagmus	-	ear pit, nystagmus, Pectusexcavatum, sparse hair	-

## Supplementary Materials

The following are available online at https://www.mdpi.com/2073-4409/9/8/1782/s1, Supplementary video S1. Zebrafish *pgap3* model displayed increased coils at 24 h post fertilization (hpf). Zebrafish locomotor behavior measurements assessed by spontaneous tail coiling at 24 hpf. We assessed the spontaneous tail coiling in zebrafish embryos at 24 hpf. (A). Un-injected control zebrafish. (B). Control_MO injected zebrafish. (C–E). *pgap3* morphants displayed increased tail coil flicking captured by The imaging source camera using the ZEISS microscope (Model Lumar 12) at a magnification of 16×. Supplementary video S2. Zebrafish locomotor behavior measurements assessed by touch-evoked swimming behavior at 72 h post fertilization (hpf). (A). Control zebrafish responds to touch stimulation with a fast swim away response to poking. (B–D). *pgap3* morphants responded differently to stimulation by swimming away with spiral swimming and circling, and after multiple stimuli, these groups became desensitized to touch stimulation in a dose-dependent behavior. Imaging using the ZEISS microscope (Model Stemi 2000-C) with a ZEISS camera (Model Axiocam Erc 5s) at a magnification of 1.6×. Supplementary video S3. 3D model of Tg:olig2:dsRed injected with *pgap3* MO at 96 h post fertilization (hpf). *pgap3* morphants displayed reduced Oligodendrocytes Precursor Cells (OPCs) expression and shorter motor axons. Transgenic representative embryo showing OPCs labeled with cytoplasmic dsRed. Morphants displayed a significant reduction in OPCs of the developing brain and optic area compared to controls and showed shorter spinal motor neuronal axons. Representative model of control and morphant zebrafish MO_G2 (moderate phenotype) and MO_G1 (severe phenotype).
